# Systematic Medical Appraisal, Referral and Treatment (SMART) Mental Health Programme for providing innovative mental health care in rural communities in India

**DOI:** 10.1017/gmh.2015.11

**Published:** 2015-07-29

**Authors:** P. K. Maulik, S. Devarapalli, S. Kallakuri, D. Praveen, V. Jha, A. Patel

**Affiliations:** 1 Research & Development, The George Institute for Global Health, 219-221 Splendor Forum, Jasola District Centre, New Delhi, India; 2 Research & Development, The George Institute for Global Health, Hyderabad, Telengana, India; 3 The George Institute for Global Health, New Delhi, India; 4 The George Institute for Global Health, Sydney, New South Wales, Australia; 5 The George Institute for Global Health, Oxford University, Oxford, United Kingdom

**Keywords:** global mental health, mental health services, mobile based healthcare delivery, low and middle income country, intervention, stigma, common mental disorders.

## Abstract

**Background.:**

India has few mental health professionals to treat the large number of people suffering from mental disorders. Rural areas are particularly disadvantaged due to lack of trained health workers. Ways to improve care could be by training village health workers in basic mental health care, and by using innovative methods of service delivery. The ongoing Systematic Medical Appraisal, Referral and Treatment Mental Health Programme will assess the acceptability, feasibility and preliminary effectiveness of a task-shifting mobile-based intervention using mixed methods, in rural Andhra Pradesh, India.

**Method.:**

The key components of the study are an anti-stigma campaign followed by a mobile-based mental health services intervention. The study will be done across two sites in rural areas, with intervention periods of 1 year and 3 months, respectively. The programme uses a mobile-based clinical decision support tool to be used by non-physician health workers and primary care physicians to screen, diagnose and manage individuals suffering from depression, suicidal risk and emotional stress. The key aim of the study will be to assess any changes in mental health services use among those screened positive following the intervention. A number of other outcomes will also be assessed using mixed methods, specifically focussed on reduction of stigma, increase in mental health awareness and other process indicators.

**Conclusions.:**

This project addresses a number of objectives as outlined in the Mental Health Action Plan of World Health Organization and India's National Mental Health Programme and Policy. If successful, the next phase will involve design and conduct of a cluster randomised controlled trial.

## Background

Providing mental health care in low resource settings is a challenge that is being faced by most regions of the world. Globally, there have been numerous calls to raise awareness of this issue and identify solutions to the problem (World Health Organization, 2001; Saraceno, 2004; Eaton *et al*. 2011). A key concern is the imbalance between the burden of mental and behavioural disorders and self-harm, which account for 9% of total disability adjusted life years (Murray *et al*. 2012), and the availability of trained mental health professionals and resources. In low and middle income countries (LMICs), the problem is particularly acute (Maulik *et al*. 2013), making it imperative to develop new strategies to deliver high quality mental healthcare in such settings.

In India, the prevalence of common mental disorders (CMDs) – depression and suicidal risk, emotional stress and anxiety disorders – vary between 13 and 50% in the community (Gururaj *et al*. 2005), but the number of trained mental health professionals are very few (World Health Organization, 2011*a*). The *treatment gap*, defined as the gap between the prevalence of mental illness and the proportion of affected individuals accessing adequate health services, is large – estimated to be 75–85% in low resource countries such as India (The WHO World Mental Health Survey Consortium, 2004). The reasons for this gap are numerous, but include poor awareness, stigma, social beliefs, as well as lack of trained mental health professionals and services (World Health Organization, 2001; Thornicroft *et al*. 2009). In India, this gap is greatest in rural regions of the country (Armstrong *et al*. 2011), where the existing primary healthcare workers in the government system are not suitably trained to identify or manage mental disorders.

### Using the primary health care system in rural India to provide mental health care

India has a three-tiered system of primary health care delivery model within the government sector. At the bottom of the pyramid is the sub-centre, which caters to 3000–5000 population and is staffed by a nurse and paramedical staff. Above that level is the primary health centre (PHC), which caters to 20 000–30 000 population and is staffed by nurses, paramedical staffs and one trained physician. It is equipped to provide basic health care and is often the focal point for most rural public health projects. Above that is the community health centre, which caters to 80 000–120 000 population. The community health centre has some specialised health care facilities and is staffed by a number of doctors, nurses and paramedical staff. In addition, the government contracts accredited social health activists (ASHAs) in each village to cater to a population of about 1000 individuals. ASHAs are recruited by the local village-level government (Panchayat) and are women residents of the village who are generally educated to grade 8–10 level. They are provided basic training in health services delivery and their primary role is to support the government programme around maternal and child health on a part-time contractual basis. They do so by making regular household visits and ensuring that the mothers and their children receive government approved care. A key role is to identify pregnant women and ensure institution-based delivery at the PHCs, for which they are incentivised. They generally work for 2–3 h per day, and use their remaining time to do their personal work. Many use this time in an opportunistic manner and provide support to other health-related activity after receiving additional training. Trained mental health professionals are available at the secondary care level district hospitals, to which any of the primary care level health facilities can refer patients.

One potential way to help bridge the mental health care gap in rural areas is to utilise the services of ASHAs. With adequate training, it may be possible for ASHAs to screen for CMDs, refer them to an appropriately trained and supported PHC doctor, assist in treatment adherence and potentially provide some education to ensure psychological well-being. However, such an approach requires innovative strategies for training and decision support tools for all relevant healthcare workers.

### Using innovative mHealth strategies to provide affordable mental health care at primary level

In contrast to the deficiency in mental health resources, India has the second highest number of mobile phone users in the world, at more than 900 million (Cellular Operators Association of India, 2014), with almost a third of such users based in rural areas. With the cost of mobile phones dropping, many more users are expected in the near future, and the potential value of using mobile technology to provide healthcare is now widely recognised (Press Information Bureau, Government of India, 2012). However, the potential benefits of mHealth solutions are likely to be dependent on a range of factors, such as availability of applications that are evidence-based and user friendly; using technology to complement existing workforce capacity through decision support tools that can be used in rural or remote communities with few trained health professionals; and training of non-physician health workers in using such tools.

The evidence-base for the effectiveness of mHealth initiatives and task-shifting strategies is currently limited. In a recent systematic review on use of mobile technology in healthcare, modest benefits on process outcomes were observed, but the effects on clinical outcomes had not been established (Free *et al*. 2013). Another broad strategy for delivering affordable healthcare in rural settings is task-shifting – where a lower-level health worker is trained to perform some of the functions that would normally be provided by physicians, without recourse to any formal education. A recent systematic review on task-shifting indicates some evidence of the effectiveness of such a strategy, but again, the evidence-base is small and currently unreliable (Joshi *et al*. 2014). Another review, assessing the use of non-specialist health workers in providing mental health care in LMICs, found some evidence to support beneficial effect on management of depression (van Ginneken *et al*. 2013). The potential benefits of an electronic clinical decision support in managing a number of health conditions, including mental health, have also been identified, particularly for tools with evidence-based algorithms that provide individualised advice at the point of care (Kawamoto *et al*. 2005; Souza *et al*. 2011).

In light of the large burden of mental illness and lack of mental health professionals, innovative solutions for the delivery of essential mental healthcare are required. Taking advantage of a large potential alternative workforce and the vast numbers of mobile phone users in India, we have developed one such solution that leverages the potential of mobile technology to provide evidence-based care for CMDs among rural communities.

### Addressing stigma against mental health

Even if such innovative solutions can be developed and implemented, the chances of success are likely to be heavily disadvantaged by poor mental health awareness and high levels of stigma associated with mental illness in the community (Gururaj *et al*. 2005). Earlier studies from rural India have demonstrated stigma against people with mental illness, both in the community as well as village health workers (Kermode *et al*. 2009), and increasing knowledge about mental health among the community and primary health workers has been suggested as a key component in delivering mental health services in rural settings (Saraceno *et al*. 2007). While no research is available from India about effective interventions to reduce stigma, studies done in other countries indicate direct social contact with people with mental illness and social marketing as effective measures (Thornicroft *et al*. 2008). In addition, family-focussed and recovery-oriented programmes have been suggested as having particular potential to address stigma in India (Koschorke *et al*. 2014).

In the proposed study, we will implement and evaluate a mobile-based intervention in the state of Andhra Pradesh in southern India, that involves task shifting and training of health workers in primary care. Our main objective is to establish the feasibility and acceptability of the intervention, as well as provide preliminary evidence about potential effectiveness in a large pilot study.

## Method

### Study sites

The study will be conducted in the West Godavari district of the state of Andhra Pradesh. It will be conducted across 42 villages. The two main sources of livelihood in these villages are farming and fisheries. The study will be conducted across two sites. The first site (*Site 1*) will be a set of 12 villages randomly selected from a list of villages in the catchment area of three PHCs, with a total population around 40 000. The second site (*Site 2*) will be a set of 30 villages from designated scheduled tribe areas randomly selected from a set of villages in the catchment of two PHCs, with a total population around 12 000. The PHCs were selected based on convenience determined by study logistics. *Site 2* involves a more disadvantaged community with fewer health resources and less access to healthcare facilities. Differences in the intervention and approach to evaluation between these sites are described in Table 1.

**Table 1.** Differences between Site1 and Site 2

	Site 1	Site 2
Setting	12 villages with a mean population of around 4000/village	30 villages with a mean population of about 400/village; villages are based in designated scheduled tribe areas
Population screened	~40 000	~12 000
Total study period	5 years	1.5 years
Intervention phase	12 months	3 months
Quantitative evaluation of anti-stigma campaign	Yes (in two villages)	No
Outcomes	Both quantitative outcomes and process indicators	Mainly process indicators

### Study duration

The duration of the intervention in *Site 1* will be 1 year, and that in *Site 2* for 3 months.

### Study population

The study will involve all eligible adults ≥18 years of age who consent to participation, are able to understand the questions and instructions, are not limited by any severe physical disorder from accessing mental health services, and who will be residing in the location for the duration of the study.

### Ethics approval

Ethics approval was received from the Centre for Chronic Disease Control, New Delhi, India.

### Study design and objectives

**Fig. 1.**
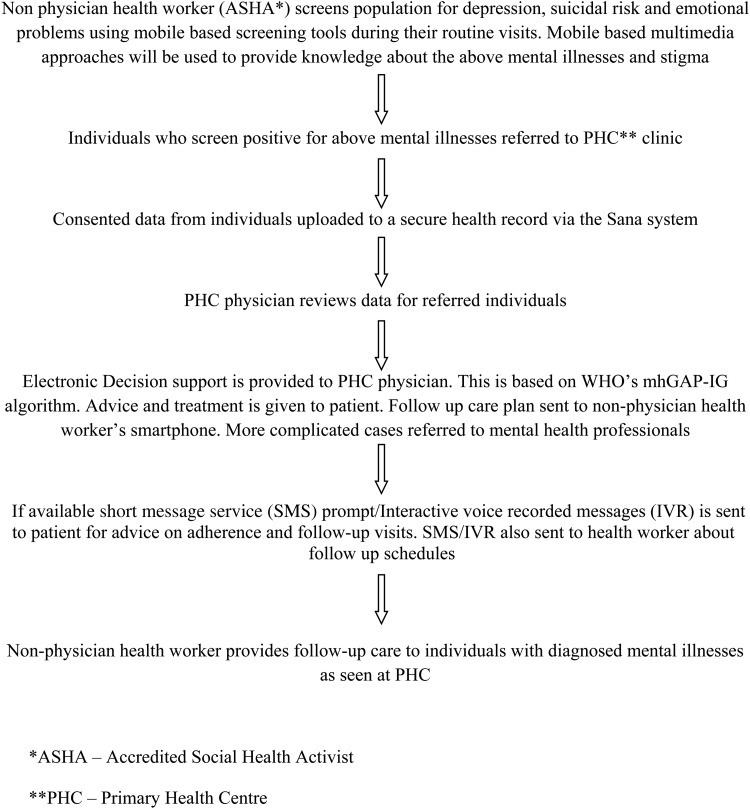
Outline of the intervention.

The project has two key objectives:

(1) Development of a multifaceted programme that includes an anti-stigma campaign and the systematic medical appraisal, referral and treatment (SMART) mental health intervention (Fig. 1).
(2) Demonstrating feasibility, acceptability and effectiveness of the programme using mixed methods.

Objective 1. Development of a multifaceted programme that includes an anti-stigma campaign and the SMART mental health intervention.

*Anti-stigma campaign*. This will involve a mental health awareness campaign based on literature and a formative research in the community. The formative research will involve focus group discussions (FGDs) with the community members and health workers and in-depth interview with a PHC doctor. The campaign will involve engagement with the community and health workers and other stakeholders through community meetings and face-to-face interactions. Multimedia approaches will be used to share knowledge about mental illness and stigma and involve local theatre performances with mental health themes and video presentations of patient and their care-givers experiences. A formal assessment will be done prior to and following the intervention in *Site 1*. It will include questions on stigma against mental disorders based on the Barriers to Access to Care Evaluation: Treatment Stigma Subscale (BACE-TS version 3) (Institute of Psychiatry, King's College London, 2011) and mental health knowledge, attitude and behaviour based on the questionnaires being used in another study (Lund *et al*. 2012). Additionally, qualitative FGDs with the community members and health workers will be undertaken to understand process related factors and acceptability of the methods used. In *Site 2*, there will be no formal assessment of stigma, but the campaign will be implemented.

*SMART Mental Health Programme.* The initial phase of the study will consist of developing the tools needed for the multifaceted intervention and for training of staff and health workers. This phase will involve developing clinical tools, the survey questionnaire, mobile-based applications and training materials for the ASHAs and doctors. Both the ASHAs and doctors will be provided training about CMDs and will be trained on the mobile-based tools for screening, diagnosis and treatment. Initial training will be followed by booster training sessions during the study period. Training will be provided by the research staff. While detailed training tools have been developed for ASHAs, the doctors will be trained using the mhGAP-IG (World Health Organization, 2011*b*).

The intervention will be implemented for 12 months in *Site 1* and for 3 months in *Site 2*. SMART Mental Health will allow primary health workers to collect patient information for screening and healthcare purposes via a tablet using the Android platform. The application will upload this information to a secure server for the doctor to review using Open Medical Record System (www.openmrs.org) – a secure, community-developed, open source, electronic medical record system platform. This will enable both ASHAs and PHC doctors to contribute to the record. ASHAs can make electronic referrals to the PHC doctor and doctors can notify the health worker via his/her tablet of the diagnosis and management plan. The applications will be tested and developed in the local Telugu language.

ASHAs will inquire about mental health status using the Patient Health Questionnaire-9 Item (PHQ9) (Pfizer Inc., 2013*a*, *b*) and Generalized Anxiety Disorder-7 Item (GAD7) (Pfizer Inc., 2013*b*, *c*). Both these tools have been validated in India and translated into Telugu, the local language (Kochhar *et al*. 2007). A score ≥10 on either PHQ9 or GAD7 will be considered as indicative of depression while screening the community, and such individuals will be referred to the PHC doctor for further clinical diagnosis and treatment. The ≥10 cut-off score has a sensitivity and specificity for major depression of 88% each, and a positive likelihood ratio of 7.1 (Kroenke & Spitzer, 2002). While a score of 5–9 is also indicative of depression, anyone with that score will be followed up again by the ASHA within 2 weeks and only those above the cut-off score of ≥10 will be referred to the PHC. Based on prior research (Gururaj *et al*. 2005), about 15% of the community could suffer from CMD, and such numbers could be managed at the PHC over the study period. The key CMDs being targeted are depression, suicidal risk and unexplained emotional and medical complaints, which are often manifestations of stress. Since individuals with depression can manifest anxiety, both depression and anxiety scales are being used to screen for depression or stress. Somatic complaints and suicidal thoughts or actions are important manifestations of depression and stress and would be assessed for in this study too.

The doctor will be using the mhGAP-IG (World Health Organization, 2011*b*) for diagnosis and management of three conditions as per the document – *depression, suicidal intent/self-harm and other emotional or medically unexplained complaints*. Primary care physicians will be trained to diagnose and manage these three conditions using a mobile-based mhGAP-IG tool. Additional knowledge would be provided about some key symptoms, such as psychotic features, and comorbid conditions, such as drug and alcohol use, which, if identified, would lead to referral to the next level of care, where trained mental health professionals are available.

The diagnosis and treatment advice provided by the doctors will then be shared with the ASHAs through their mobile devices, such that the ASHAs can follow up on the individuals and ensure treatment adherence. Interactive voice recorded messages will also be sent to the health workers and community members who have mobile phones about treatment adherence, steps in facing stress and other mental health well-being tips using pre-determined algorithms.

All electronic data being uploaded by ASHAs and doctors will be used to monitor their activity by the project manager. Continuous support will be provided on a regular basis and trouble-shooting will be done as per need. This will be supplemented by analytics embedded within the system which will provide reports on how ASHAs and doctors actually used the system. The doctors would be provided contacts of local psychiatrists/psychologists, available in the nearest towns, whom they could contact to discuss difficult cases and also refer such cases, if needed.

Objective 2. Demonstrating feasibility, acceptability and effectiveness of the programme using mixed methods.

The intervention will be evaluated in a single cohort using pre- and post-intervention comparisons. Both prior to the intervention and following it, detailed evaluation will be done by trained interviewers on different components of the intervention, using mixed methods approach. Baseline data on CMD prevalence and stigma will be collected from all eligible consenting adults; ≥18 years in all villages across both sites. Post-intervention data will be collected from only the screen-positive individuals. All data will be analysed using appropriate statistical techniques and both univariate and multivariate analyses will be done.

FGDs and in-depth interviews will be conducted following the intervention with the community participants, ASHAs and physicians about the intervention and its advantages and disadvantages and potential steps to improve them. Interviews will be semi-structured and conducted by researchers with a practical working knowledge of the settings in which the tool will be implemented. Interview recordings will be professionally transcribed, translated to English (where necessary) and thematic content analysis will be conducted. Interview transcripts will be reviewed contemporaneously with data collected and subsequent interviews will be refined on the basis of the preliminary findings. This process will require the research team to regularly meet and agree on the key themes arising from the data.

### Key outcomes

The key outcomes of the study will be the following:

(1) Quantitative data will be assessed for the following outcomes:
  • Prevalence of CMD in the community.
  • Proportion of individuals who have accessed the health system for management of a mental disorder in the previous 12 months.
  • Proportion of individuals who had an ASHA-administered PHQ9 and GAD7.
  • Proportion of individuals appropriately referred by the ASHA to the PHC doctor.
  • Proportion of individuals appropriately referred by the PHC doctor for more specialised care.
  • Proportion of individuals appropriately prescribed anti-depressant or anxiolytic treatment.
  • Proportion of individuals with a PHQ9 and/or GAD7 score ≥10 among those who have accessed the health system for management of a mental disorder in the previous 12 months.
  • Proportion of people in the community who show an improvement on the stigma scores and mental health knowledge scores after the stigma awareness campaign.
  • Proportion of screen-positive people who accessed mental health services and felt stigmatised.
  • Proportion of community members and health care workers who show an increased attitude and knowledge about CMDs.
(2) Qualitative data will also be collected and the key questions that will be explored include:
  • How ASHAs use the intervention?
  • What effects it has on doctor practices?
  • What are participant experiences of receiving the intervention?
  • What are the stigma related barriers to seeking care?
  • What are some important steps that can help in reducing stigma?
  • What are some of the stigmatising experiences of those who accessed mental health care?

### Sample size calculation and data analyses

Using conservative estimates of adult CMD prevalence (Gururaj *et al*. 2005) and 75% consent rate, we anticipate between 3000 and 4000 adults in *Site 1* will be suffering a CMD. Earlier study that focussed on provision of mental health services in India using primary care workers had found an intraclass correlation (ICC) of 0.03 (Patel *et al*. 2011). Given that this study has a behavioural intervention, we are assuming an ICC of 0.1.The expected number of adults with CMD in our study in *Site 1*, has 80% power at two-tailed *α* = 0.05, to detect a 20% increase in mental health services use accounting for clustering (ICC 0.1).

At *Site 2*, the proportionate change in mental health service use from baseline will also be assessed. However, there will be insufficient power to reliably evaluate the effects on quantitative outcomes. Therefore, feasibility will be primarily evaluated using qualitative data.

Descriptive statistics will be conducted and data would be checked for consistency. Appropriate univariate and bivariate analyses would be undertaken and tests of significance would be conducted. Suitable regression models will be used to account for confounders. The models would be finalised based on techniques to identify the most appropriate model that explains the variability. Repeated measures data would be based on longitudinal data analyses principles.

Qualitative data will also be analysed to identify recurring themes and key concepts generated during the qualitative interviews. Interviews will be semi-structured and conducted by researchers with a practical working knowledge of the settings in which the tool will be implemented. Interview recordings will be professionally transcribed, translated to English (where necessary) and thematic content analysis will be conducted.

## Conclusion

The SMART Mental Health Programme is responsive to the Mental Health Action Plan of World Health Organization for the year 2013–2020 (World Health Organization, 2013). The four objectives of the Mental Health Action Plan are: to strengthen effective leadership and governance for mental health; to provide comprehensive, integrated and responsive mental health and social care services in community-based settings; to implement strategies for promotion and prevention in mental health and to strengthen information systems, evidence and research for mental health. In order to fulfil these objectives, it is proposed that high quality, culturally appropriate research be carried out globally which will help to provide evidence-based mental health services and knowledge that leads to better mental health outcomes.

The programme will evaluate a novel approach to the provision of evidence-based mental health care in particularly disadvantaged communities. This piece of implementation research is designed to be responsive to the end users responsible for the provision of essential primary healthcare. While the current study will only provide preliminary evidence of likely effectiveness, its successful completion will be crucial to refine the intervention and inform a more definitive large cluster randomised trial in the future. Such a study will be designed to provide robust evidence on effectiveness and cost-effectiveness.

The beneficial effects of initiatives such as this on mental health at a global level are potentially substantial. This project will add to the experience of another project being conducted in Kenya (http://www.grandchallenges.ca/grantee-stars/0414-01/) where primary care workers are using the mhGAP tool to screen 1000 patients attending four primary care sites. While the project uses a mobile-based system, it does not include a community-based screening, and does not link village health workers with clinic-based advice to enable follow up and adherence (Christine Musyimi, personal communication, http://www.grandchallenges.ca/grantee-stars/0414-01/, Principal Investigator). It also is focussed only on depression, unlike this study, which includes CMDs. While the promises of mHealth initiatives in LMIC are substantial, to date this promise has not yet been realised at scale. In this regard, the SMART Mental Health initiative is important as it goes beyond the technology platform in taking a community and systems-based approach to the delivery of healthcare. However, rigorous evaluation to not only determine whether the intervention works, but identifying populations and context in which it works better and how it can be developed further are also important. Lessons learned from this project will not only inform mental health service delivery in LMICs, but also geographically inaccessible and low resource settings of high income countries, where more specialised services may not be readily available. The mHealth based care may also have lessons for urban areas and areas with better facilities too, where suitably adapted mobile-based mental health service delivery catering to specific populations can be developed using the principles of this study.

The SMART Mental Health Programme is essentially a primary care based model so it does add to the second objective of World Health Organization's Mental Health Action Plan of developing integrated and responsive community-based mental health services. This is also in line with the objectives of India's National Mental Health Policy and National Mental Health Programme, as both emphasise community-based mental health services that are based on evidence-based practices (Ministry of Health and Family Welfare, Government of India, 1982, 2014). This is also consistent with growing global awareness and calls for using primary health care workers in providing mental health care (Kakuma *et al*. 2011). Task shifting and training primary care workers has shown to be effective for some health conditions (Joshi *et al*. 2014), but the evidence for mental health services is still unclear, and this programme should give an opportunity to add some evidence towards that objective and provide valuable information about delivering primary care based mental health services in low resource settings. The lessons learned may be applicable to other regions within India and also other countries facing similar problems as India, with suitable local and cultural adaptation.

Finally, stigma plays a key role in preventing people with mental disorders from accessing appropriate services (Clement *et al*. 2014). Stigma also has a role in promotion and prevention of mental disorders. Combating stigma is one of the objectives of the Mental Health Action Plan. This study will address the issue of stigma and will evaluate the strategy used. We hope that lessons learned will help to develop better strategies to reduce stigma in similar communities across India and other countries. Again, this is consistent with the overarching vision of both the National Mental Health Programme and Policy of the Government of India, so it would be of direct relevance to provision of mental health care in India, if found to be effective and scalable.

In conclusion, SMART Mental Health Programme will address a number of objectives that form part of World Health Organization's Mental Health Action Plan and policies and programmes of the Government of India. The results may have implications for not only the community in India, but also similar low resource settings in other parts of the world. If found successful, suitably adapted translational research based on the lessons learned from this study could help other countries to modify their mental health service delivery models.
